# Intravascular endoscopy improvement through narrow-band imaging

**DOI:** 10.1007/s11548-017-1579-4

**Published:** 2017-03-30

**Authors:** Axel Boese, Akhil Karthasseril Sivankutty, Alfredo Illanes, Michael Friebe

**Affiliations:** Chair for Catheter Technologies, Otto-von-Guericke University, Universitätsplatz 2, 39106 Magdeburg, Germany

**Keywords:** Narrow-band imaging, White light imaging, Arterial wall, Vascular endoscopy, Vascular imaging, Stroke, Aneurysm, Stenting, Stenosis, Vessel wall defects

## Abstract

**Purpose:**

Recent advances in endoscopy have led to new technologies with significant optical imaging improvements. Since its development a few years ago, narrow-band imaging (NBI) has already been proved useful in detecting malignant lesions and carcinoma in clinical settings of urology, gastroenterology and ENT. The potential of this technology for imaging applications of the arterial vessel wall has not been properly analysed yet, but with the observed benefits could prove valuable for this clinical use as well.

**Methods:**

In order to assess the efficacy of NBI, defects such as burns and mechanical tears were created on the walls of an arterial vessel sample. Ex vivo imaging using NBI and white light imaging (WLI) were performed with rigid and flexible fibre endoscopes.

**Results:**

A thorough comparison of the images proved that NBI enhances the visualisation of lesions and defects on the artery walls compared to normal WLI.

**Conclusion:**

WLI provides a direct image of the vessel lumen and its anatomical shape. It is suitable for observation and documentation of intravascular therapies. NBI images are more distinct and have more contrast. This helps to detect even small defects or changes on the inner vessel wall that could provide additional information and lead to more precise and personalised therapies.

## Purpose

Through technology advancements and the introduction of complete new methodologies, endoscopic intravascular visualisation is becoming more and more interesting as a diagnostic tool. Intravascular imaging could be used to identify conditions that lead to strokes, aneurysm and embolism. In vivo imaging of the arteries could additionally help to find calcifications [1], plaques [2, 3], thrombus [4] and also to assess the placement of stents [5]. Thus intravascular imaging could play an important role in the clinical decision making process and provide therapy relevant information. Several studies have been conducted using X-ray-based angioscopic systems to illustrate and record the changes that are associated with vascular diseases in various arteries [6]. New hybrid intravascular imaging technologies have been developed by combining, for example, optical coherence tomography (OCT), intravascular ultrasound (IVUS), near-infrared spectroscopy (NIRS), Raman spectroscopy, intravascular photoacoustic imaging (IVPA) and near-infrared fluorescence (NIRF). Bourantas et al. presented a nice work illustrating these hybrid technologies [7]. NBI is one method of image enhancement [8] commonly used in endoscopic imaging. Comparisons on the performance of NBI and WLI have already been made in urology [9] and gastroenterology [10, 11]. It was shown that NBI can selectively improve the contrast of blood vessels or haemoglobin containing structures. Most of the components of the vessel wall do not present any colour. Just in case of defects in the wall, haemoglobin is accumulating. This can be detected using NBI. In this paper, we present a study on the feasibility of NBI in intra-arterial wall visualisation and compare the acquired images with those obtained using conventional WLI endoscopy and microscopy.

NBI uses blue (415 nm) and green (540 nm) wavelength light to highlight features from the mucosal surface that are not typically seen using WLI. The shorter wavelength can penetrate only the superficial layers while the longer one can penetrate deeper. These wavelengths are used since the haemoglobin absorption of light is there at a maximum. The surface capillaries usually appear brownish and the deeper vessels appear cyan in NBI image. The NBI technology is incorporated into the endoscopic image system (Olympus, Hamburg, Germany) and can be activated by pressing a single button, making it quite convenient for the endoscopist to switch between WLI and NBI.

## Methods

### Sample preparation

The tests were done in our catheter laboratories (INKA—Intelligent Catheter, OVGU, Magdeburg). In order to make the comparison, certain conditions had to be created on the tissue samples to simulate mechanical and thermal ablation defects, or inflammations. Freshly extracted porcine vessels were used for the first experiments (femoral, aortic and carotid arteries). To find out the right conditions for creating a change in the inner vessel surface, a few tests were done initially and for that the samples were opened up. Acids including acetic acid and sulphuric acid did not yield perfect results for simulating inflammations. Heat was identified to mimic ablation defects as known from cardiac procedures. Burning the samples shortly, using a solder at $300\,^{\circ }\hbox {C}$ created distinct burns on the tissue surface. Scalpels were used to create small mechanical defects on the inner tissue surface to simulate dissections. Usually dissections are caused by weak artery walls or as a result of vascular interventions (e.g. stent-triver or vascular implantations). Other causes could include genetic defects, accidents, twisting, choking, smoking and hypertension [12]. Vascular dissections can lead to the development of aneurysms. Also, an accumulation of blood on the artery wall could result in thromboses or clots and with that potentially could cause a stroke.

The preparation of the samples was done under microscopic guidance (SZ61 stereo microscope Olympus, Germany).

### Test set-up

**Fig. 1:**
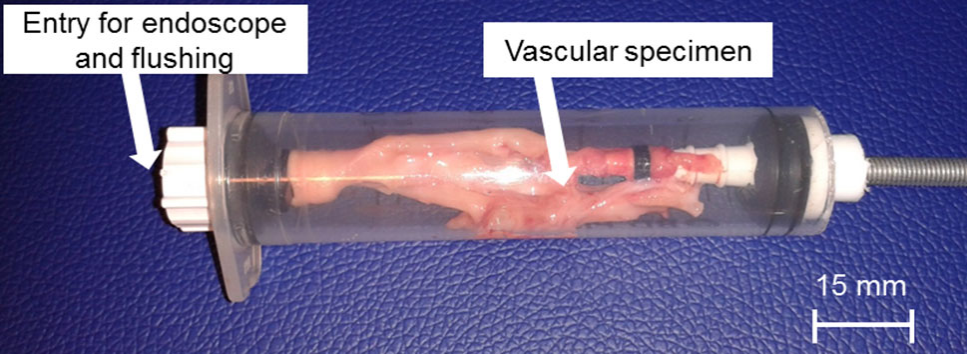
Tissue holder with vascular specimen, one side open for flushing and introducing the endoscope

**Fig. 2:**
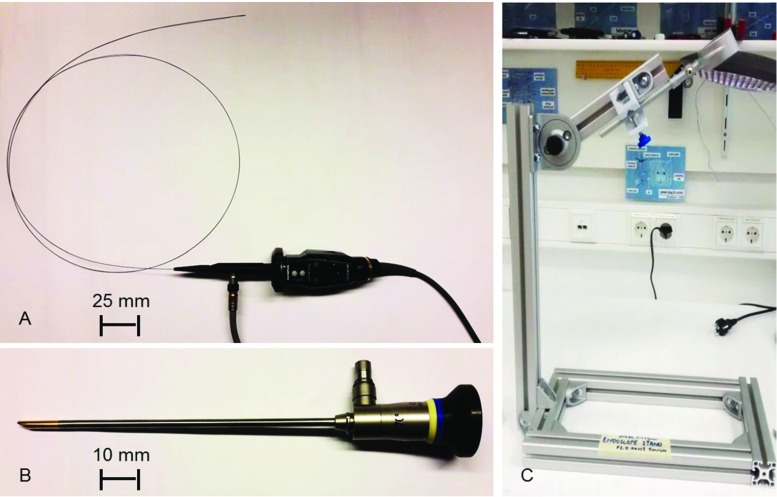
Test set-up for image acquisition; **a** flexible fibre endoscope; **b** rigid 4-mm endoscope; **c** stand to hold the endoscope and tissue holder depending upon distance and angle

For the test system, a tissue holder was created based on a 50-ml syringe, tube connectors and plugs for sealing (Fig. 1). The tissue holder provides an entry for the endoscope and convenient flushing. The tissue sample were pulled over the tube connectors and fixated. By increasing the distance of both connectors inside the syringe, the sample can be stretched for image acquisition. A stand to hold the endoscope was also mounted using standardised alloy profiles (ITEM, Germany) (Fig. 2c). After fixation of the camera and endoscope on the stand, the tip of the endoscope can be fed into the tissue holder under defined conditions. Images were obtained with a standard endoscopic imaging system (EVIS EXERA III + CH-S190-XZ camera module, all Olympus, Germany).

For the first prove of concept, a rigid 4-mm endoscope was used (WA96205A $70^{\circ }$ autoclave rigid scope 4 mm $\varnothing$, Olympus, Germany) (Fig. 2b). This endoscope offers high image quality but cannot be placed into the vasculature in clinical routine.

A flexible endoscope fibre (Prototype, $\varnothing = 0.5\, \hbox {mm}, {L}= 1500\, \hbox {mm}$, KARL STORZ, Germany) was used for image acquisition under more realistic conditions (Fig. 2a). The fibre endoscope can be placed inside a catheter that provides access to the vasculature. Image quality is poor due to the low number of fibres (5000 pc).

### Acquisition of resulting images

The sample for the final test was a fresh extracted portion of the iliac artery obtained from a swine. It was kept in blood after extraction because the presence of haemoglobin inside the tissue is necessary for NBI imaging. Before doing the final examination, lesions were created using the solder and by the scalpel. The tissue sample was then inserted into the tissue sample holder. Subsequent flushing was done using a syringe with blood and saline solution for cleaning. The rigid 4-mm endoscope was inserted into the sample and imaging using WLI and NBI was performed. Later the sample was dissected and viewed under the microscope.

Smaller specimens of porcine vasculature $({>}{3}\,\hbox {mm}\,\varnothing$) were imaged with the 0.5-mm fibre endoscope. Mechanical defects were applied on the intima layer using a 21G needle. A defined placement of burns was not possible in these trials. The specimens were filled with blood first and flushed with saline solution for imaging. Images of mechanical defects and vascular bifurcations were obtained after flushing.

### Image analysis

For comparison of the images captured with WLI and NBI an image analysis was performed. A comparable region of interest (ROI) covering the lesion and parts of the surrounding tissue was selected in both associated images (WLI and NBI). For the ROI of the extracted sub-images the line profile histogram of every pixel row and the gradients of these line profiles were computed. The results were plotted into intensity change diagrams. The average of intensity change was computed and compared for WLI and NBI.

## Results

**Fig. 3:**
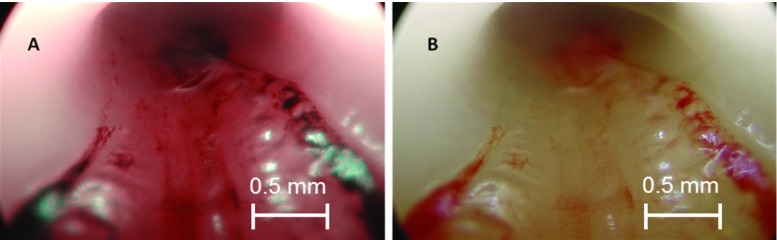
Endoscopic images of the vessel lumen. **a** Narrow-band image of the iliac artery sample after being flushed with blood. **b** White light endoscopic image of the same portion

WLI and NBI images of the iliac artery—after the lumen was flushed with saline solution to remove blood—are shown in Fig. 3. The NBI image showed more contrast when compared to the WLI image. The light absorption of the haemoglobin is enhancing the surface structure of the inner vessel lumen.

**Fig. 4:**
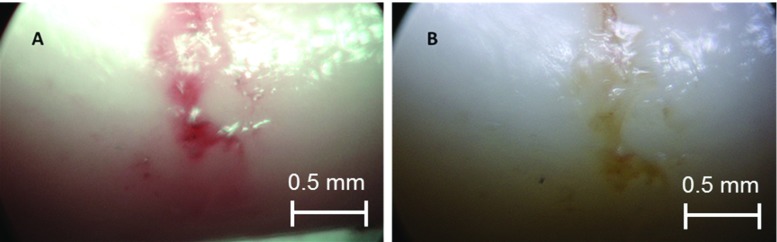
Laceration induced by the scalpel. **a** Narrow-band image showing a more prominent lesion. **b** White light endoscopic image appears to have comparatively less contrast

**Fig. 5:**
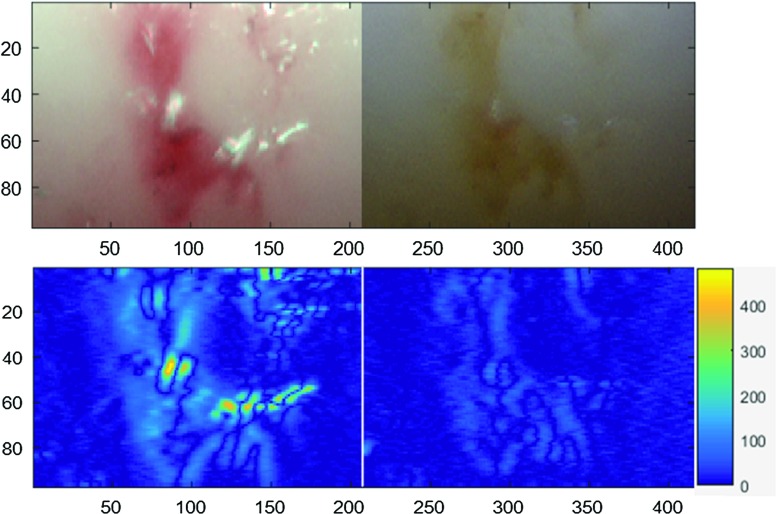
Change in intensity calculated by image analysis for the mechanical defect: *upper row* selected ROI in NBI and WLI, *lower row* calculated gradients of intensity changes for NBI and WLI, NBI showed an increase of gradients of 19% in average

The mechanical defect (identical to a case of arterial dissection) induced by scalpel on the intima layer was recognisable in both images (Fig. 4). When compared, the boundaries of the laceration on the surface were more distinct in the NBI image due to the better contrast. The difference in the wavelengths used made the injury look red in narrow-band image and yellow in the white light image. Figure 5 shows the change in intensity calculated in image analysis. NBI showed an increase of intensity gradients of 22% in average, leading to the visible enhancement of the structure in NBI compared to WLI. More and smaller details of the defects are distinguishable in NBI. The enhancement leads to a three-dimensional impression. These findings were confirmed by a clinical partner.

**Fig. 6:**
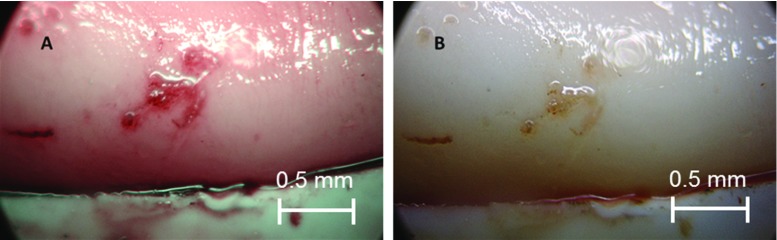
Endoscopic image of the burned portion of the intima surface. **a** Narrow-band image showing a bulged and a crimsoned injury. **b** White light image showing a comparatively obscure image

**Fig. 7:**
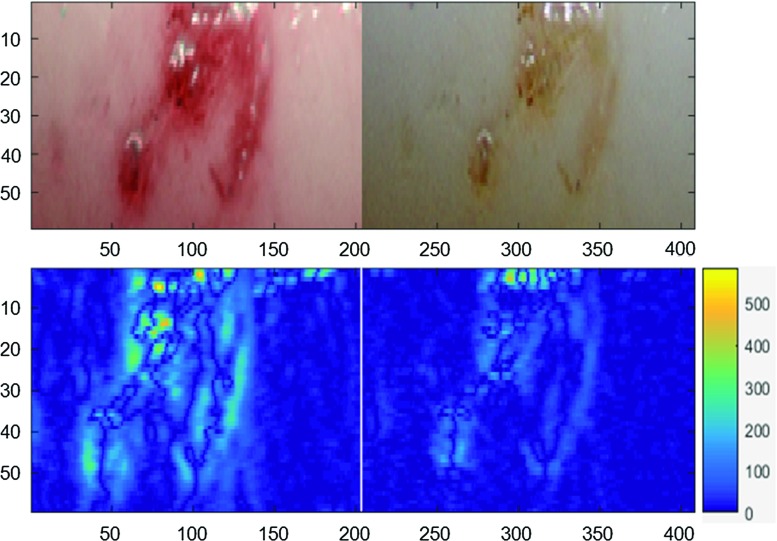
Change in intensity calculated by image analysis for the lesion created by heat: *upper row* selected ROI in NBI and WLI, *lower row* calculated gradients of intensity changes for NBI and WLI, NBI showed an increase of gradients of 22% in average

A second pathological condition was created on the sample by applying heat. The portion of the vessel which was scorched by the soldering machine was well noticeable (Fig. 6). The burns were more prominent in the NBI image as the charred portion appeared to be relatively bulged and crimson under the narrow band. The WLI image also showed the injury, but the NBI image was more explicit and definite. Figure 7 shows the results of the image analysis of the selected ROI of the burned portion.

**Fig. 8:**
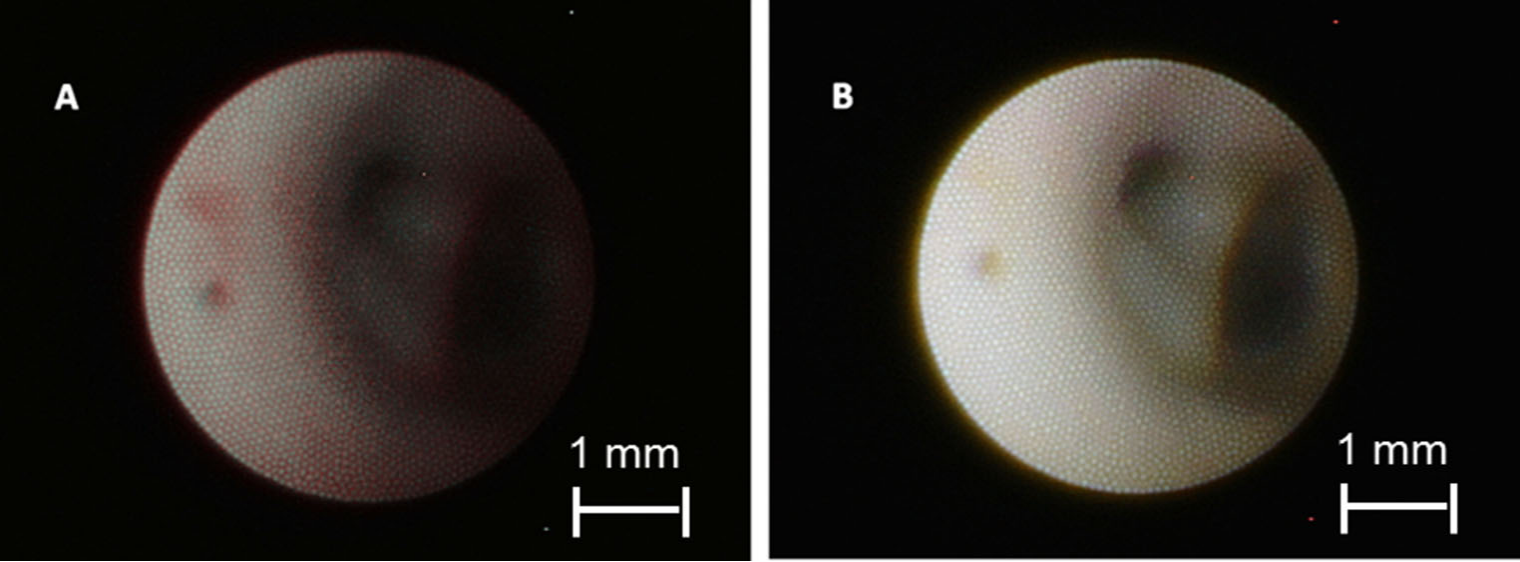
Fibre endoscopic image of a bifurcation in the artery. **a** Narrow-band image showing a red and dark image. **b** White light image showing a comparatively brighter and clear image. Lesion on the left site is more visible in NBI

The described images were obtained to prove the potential of NBI in vascular endoscopy in general. A rigid 4-mm endoscope was used providing an outstanding image quality. This endoscope cannot be used in smaller vasculature, however. For a realistic imaging scenario of these vessels, a 0.5-mm prototype fibre optic endoscope was used. Figure 8 shows a NBI compared to a WLI image. The endoscope is placed in a 3-mm specimen in front of a bifurcation. The vessel branches are clearly visible in WLI. The mechanical defect can be seen on the left side of the image. Structure and proliferation are more distinct in NBI.

## Discussion

Optical imaging technologies in general can have a lot of intravascular applications. This includes the use during pre-operative, intraoperative and post-operative procedures. The biggest benefit of optical endovascular imaging compared to other imaging technologies is the visualisation of the real vessel lumen. Additionally changes of the inner vessel surface can be detected. The presented combination with NBI addresses this potential.

Conventional white light endoscopy is already in use for intravascular imaging for special applications. NBI has been proved useful in other clinical areas such as urology and gastroenterology or ENT. In this paper, the feasibility and potential advantages of NBI was evaluated in an intravascular setting. As expected, NBI showed an enhancement of structures of interest compared to WLI imaging.

Both technologies (NBI and WLI) showed recognisable images of the injuries, made on the artery wall. NBI, with integrated optical filters, showed more contrast, however, was more intensively coloured and had clearer definite boundaries. The white light endoscopic images are sufficient to identify vascular structures such as bifurcations or aneurysms and larger and deeper injuries. NBI offers a clear advantage for detecting and analysing smaller lesions though and faster.

An additional imaging option can be beneficial for the diagnosis and subsequent therapeutic procedures and for the documentation of treatment success. Optical imaging of inner blood vessels can provide realistic information of the shape and dimensions of the vasculature that cannot be acquired by angiography [13]. Vascular endoscopy can also be used for inspection of vascular occlusions or observation of aneurysm formation. The observation and documentation of interventions, e.g. stent placement, can be another possible application [14].

But, optical vascular endoscopy is an interventional technique and with that bears a risk for the patient. Placement of the endoscope through a catheter is necessary and flushing to remove blood is essential to guarantee good image quality. For that, we propose the same procedure that is used for intravascular optical coherence tomography (OCT) [15, 16]. A short bolus of saline solution removes blood for an image sequence.

The results shown in Figs. 3, 4, 5 and 6 were acquired under optimal conditions. The rigid endoscope images were clear and distinct with even more contrast in NBI images. But the large size of the rigid endoscope limits its application inside vessel structures.

The flexible endoscope fibre is significantly smaller (0.5 mm vs. 4 mm) and can be used with a catheter to ensure vascular access and easy navigation. The images obtained with this fibre are more realistic (Fig. 8). The white light image appears to be better illuminated and is bright. The narrow-band image is relatively dim but still has better contrast and enhances even small defects (Fig.  8a).

While the defects are detectable in both WLI and NBI, comparison of the images indicates that NBI undoubtedly provides more accurate estimation of vascular lesions. NBI also produces images with a sharp contrast, thus eliminating the need of contrast enhancing dyes that would be unrealistic inside the vasculature.

NBI provides decisive information’s in guiding the decision making process for the diagnostic evaluation and for choosing the subsequent therapy options.

We presented a first trial of the use of NBI in a laboratory ex vivo vascular imaging set-up. In vivo use would have given more definite evidence as NBI needs the presence of haemoglobin inside the tissue. With that more enhanced and magnified vessel wall imaging could be possible. Also a combination with other imaging modalities to prove the findings in optical vascular imaging can be beneficial as described in [17, 18].

The biggest challenge in optical vascular imaging still is the removal of blood for a short time period. The image protocols as used for intravascular OCT could solve that issue. Also the endoscope needs to be improved to achieve higher image quality with a higher number of fibres and more flexibility. The flexibility is a major requirement for accessibility of more distal vascular structures. Further research could be done in testing the feasibility of NBI in detecting other vascular diseases such as atherosclerosis or the deviation between hard and soft plaque. More pre-clinical trials have to be performed to prove a safe workflow and the diagnostic potential of NBI in intravascular imaging.

## Conclusion

In the paper, the feasibility of NBI compared to WLI in an ex vivo intravascular setting was tested. NBI showed superior contrast and better defined representation of lesions, whereas WLI provides more information of the inner vascular structure itself. We showed that even with low-quality images acquired with an endoscopic fibre, small vascular lesions can be depicted using NBI. More trials and in vivo imaging have to be performed to develop a safe application of optical vascular imaging.
